# Changes in lipids metabolism indices as a result of different form of selenium supplementation in chickens

**DOI:** 10.1038/s41598-022-18101-2

**Published:** 2022-08-15

**Authors:** Damian Bień, Monika Michalczuk, Dominika Szkopek, Misza Kinsner, Paweł Konieczka

**Affiliations:** 1 Department of Animal Breeding, Institute of Animal Sciences, Warsaw University of Life Sciences, Ciszewskiego 8, 02-786 Warsaw, Poland; 2 Department of Animal Nutrition, The Kielanowski Institute of Animal Physiology and Nutrition, Polish Academy of Sciences, Instytucka 3, 05‑110 Jabłonna, Poland; 3 Department of Poultry Science, University of Warmia and Mazury in Olsztyn, 10-719 Olsztyn, Poland

**Keywords:** Animal physiology, Biomechanics

## Abstract

Selenium is an essential element that is important for many metabolic processes. Feed components used in chicken nutrition, especially cereals, may be deficient in selenium, hence selenium supplementation is necessary. Taking into account the progress in breeding, and thus the higher demand of birds for this element, it seems obvious to investigate an increased selenium dose in the diet of chickens. The aim of the study was to evaluate the effect of feed enriched with different forms of selenium at an increased dose of 0.5 mg/kg feed on the profile and metabolism of fatty acids in the breast muscle and liver of chickens. The study was conducted on 300 Ross 308 chickens reared for 42 days under standard conditions. The control group received feed supplemented with sodium selenite at a dose of 0.3 mg/kg feed. The research groups received different forms of selenium (sodium selenate, selenised yeast, nano-selenium) at an increased dose of 0.5 mg/kg feed. The study showed that the administration of different forms of selenium in the feed affected its concentration in the breast muscle and liver (*p* ≤ 0.01). Nano-selenium was found to have a high bioavailability, but also a lower risk of toxicity compared to other forms of selenium. Using different forms of selenium (*p* ≤ 0.01) at a dose of 0.5 mg/kg feed can significantly modify the fatty acid profile, lipid and enzymatic indices of fatty acid metabolism in breast muscle and liver.

## Introduction

In monogastric animals, unlike in the ruminants, unsaturated fatty acids (UFA) are not biohydrogenated from feed. For this reason, there is a correlation between delivered dietary fatty acids (FA) and those deposited in tissues of such animals as poultry^1,2^. A high polyunsaturated FA (PUFA) concentration in the feedstuff bears the risk of increased susceptibility of tissue lipids to oxidation, which in turn may modify both taste and aroma (rancid) and the nutritive value of meat. To prevent oxidative processes, feed mixtures for poultry are supplemented with additives featuring antioxidative properties that preserve oxidative stability and, by this means, also the oxidative balance of lipids in meat^3^. Selenium (Se) is one of the commonly applied antioxidants. It is used in animal feeding usually in two forms: inorganic—as sodium selenite (Na_2_SeO_3_) or sodium selenate (Na_2_SeO_4_; SS), or organic—as selenomethionine (SeMet) or selenocysteine (SeCys). Selenium is an essential bioelement which—together with other microelements, like zinc and iodine—plays an important role in the proper functioning, development, and growth of various organisms^4^. So far, feed mixtures for broiler chickens have mainly been supplemented with inorganic forms of Se. However, with the advance of research on Se, also its other forms have been implemented, including especially these more readily absorbed in tissues compared to SS and sodium selenate^5^. The Se mitigate oxidative stress and peroxidative damage of UFA, and affect the effectiveness of fatty acid biosynthesis in animal tissues^6,7^. Selenium deficiency in diet may adversely influence the conversion of linolenic acid (ALA) to eicosapentaenoic acid (EPA) and docosahexaenoic acid (DHA), resulting in the unbeneficial n-6/n-3 ratio in tissue lipids. Also, Se addition to feedstuffs for livestock modifies the fatty acid profile of meat^8–12^. The consumption of chicken meat increases successively each year compared to other meat types. Therefore, the enrichment of diets for poultry with appropriate forms of Se would allow producing poultry meat rich in high-quality n-3 fatty acids that are indispensable in the human diet. Recent studies have indicated that especially the long-chain n-3 PUFA play a vital role in preventing cardiovascular diseases^2,13,14^. In addition, broiler diet enrichment with Se nanoparticles has been shown to be more effective because nano-Se does not have to be metabolized before being incorporated into selenoproteins and may be absorbed by the body more effectively than SS^15,16^. Considering multiple benefits of using nanoparticles, like increased bioaccessibility and absorption^17–19^, improving the feed conversion ratio, promoting growth and development of muscle cells, improving gut microbiota, and supporting avian coccidiosis prophylaxis^16,20^, it seems likely that nano-Se can also increase the possibility of chicken meat enrichment with PUFA however, data in this regards are scarce.

This study aimed to compare the effects of various forms of Se on fatty acid metabolism and health status of broiler chickens.

## Results

**Figure 1:**
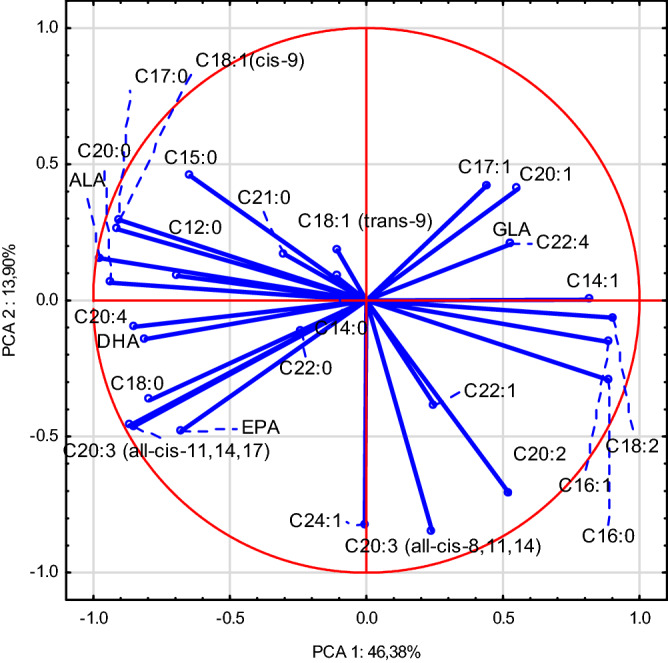
The distribution of the factor loadings of the variables of breast muscle samples, i.e. the correlations of fatty acids between the primary variables and the principal components. Linoleic acid (LA, C 18:2) was the most strongly and positively correlated with the first principal component.

**Figure 2:**
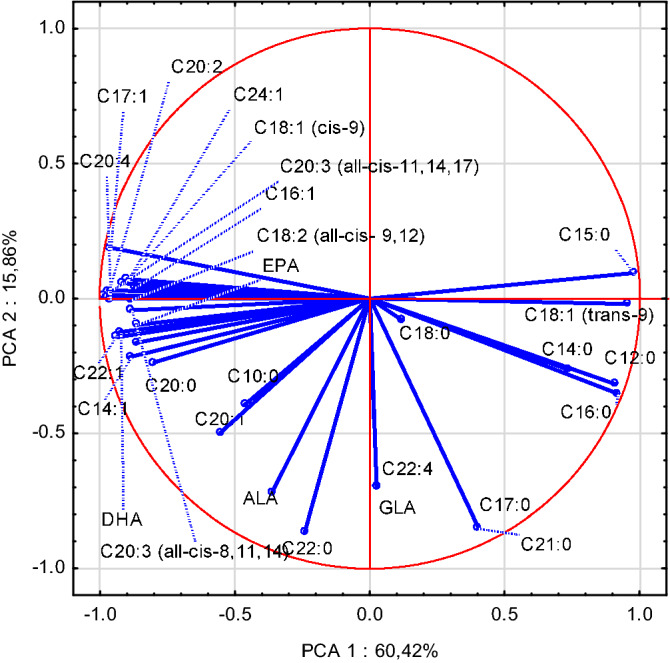
The distribution of the factor loadings of the variables of liver samples, i.e. the correlations of fatty acids between the primary variables and the principal components. Pentadecanoic acid (C15:0) was the most strongly positively correlated with PCA 1, whereas behenic acid (C22:0) was the most strongly negatively correlated with PCA 2.

The Principal Component Analysis (PCA) was employed for the tentative exploration of data collected for the samples of breast muscles (BM) and liver. The PCA performed for the samples of BM intramuscular fat allowed distinguishing six principal components with Eigenvalues above 1.0 (Kaiser's criterion) responsible for 90.68% of the total variance. The variables contributing to this grouping (having Eigenvalues higher than 0.7) included the following fatty acids (Fig. 1): C14:1, C16:0, C16:1, and C18:2 (positive values for PCA 1) as well as C17:0, C18:0, C18:1 cis-9, C20:0, ALA, C20:3 all-cis-11,14,17, C20:4, C22:5, and DHA (negative values for PCA 1). In the case of PCA 2, significant turned out to be only the negative charges: C20:2, C20:3 all-cis-8,11,14, and C24:1. Such a point distribution alongside the PCA 1 reflects the effect of using higher doses of other Se forms (T2 and T3) than SS doses used in CON and T1 diets (0.3 mg/kg and 0.5 mg/kg, respectively) on the FA profile in BM intramuscular fat. The PCA performed for liver fat samples allowed distinguishing four principal components with Eigenvalues above 1.0 (Kaiser's criterion) responsible for 90.98% of the total variance. The variables contributing to this grouping of points (having Eigenvalues higher than 0.7) for PCA 1 included the following fatty acids (Fig. 2): C12:0, C14:0, C15:0, C16:0, and C18:1 trans-9 (positively correlated) as well as C14:1, C16:1, C17:1, C18:1 cis-9, C18:2, C20:0, C20:2, C20:3, C22:1, C20:3, C20:4, EPA, C24:1 and DHA (negatively correlated). In the case of PCA 2 (13.90%), only C17:0, C18:3, C21:0, and C22:0 were negatively correlated. As observed for the samples of BM intramuscular fat, the distribution of points alongside PCA 1 reflects the influence of using other forms of Se than inorganic Se in the chicken diet, i.e. selenized yeast (SY) and nano-Se, and modification of fatty acid profile in BM and liver caused by diet supplementation with various forms of Se.

This study analyzed feed mixtures supplemented with various forms of Se in a dose of 0.5 mg/kg feed, which is upper limits of organic Se supplementation in the chicken feed. A comparison of the fatty acid profile of BM intramuscular fat and liver fat of birds from the particular experimental groups demonstrated that the administration of the increased doses of various Se forms significantly (*p* ≤ 0.01) affected the lipid composition of the examined tissues, including concentrations of SFA, MUFA, PUFA as well as n-3 and n-6 FA (Table 1). The major FA responsible for differences in the content of PUFA was C18:2 acid (LA) in BM samples and C20:4 acid (n-6) in liver samples. The use of SY or nano-Se in chicken feeding enables significantly increasing MUFA contents and increasing PUFA contents in BM The highest content of n-3 FA in the samples of BM intramuscular fat was determined in group T1, where birds received a diet with an increased SS dose. The increased content of n-3 fatty acids resulted in the lowest content of n-6 fatty acids and a lower n-6/n-3 ratio. In the case of the liver fat samples, the most beneficial n-6/n-3 ratio was determined in the CON group, which additionally featured the highest content of n-3 fatty acids. Broiler diet supplementation with SY and nano-Se significantly (*p* ≤ 0.01) increased PUFA content in BM intramuscular fat. In the case of liver fat samples, the same effect (*p* ≤ 0.01) was achieved upon diet supplementation with an Se dose of 0.3 mg/kg feed. The contents of LA and ALA fatty acids, which are significantly depended on Se form used in the broiler diet. The highest LA content in BM was determined in group T2, whereas the lowest one in group T1. The ALA content was inversely proportional to the LA content. The highest ALA content was determined in group T1 fed a diet with the increased SS dose, whereas the lowest one in groups T2 and T3. The analysis of DHA contents in BM and liver demonstrated their significantly (*p* ≤ 0.01) higher content in the groups of chicken broilers administered the SS-supplemented diet.

**Table 1:** The effect of using increased doses of various forms of selenium on the fatty acid profile of chicken broiler breast muscle and liver.

Indices	Breast muscle						Liver					
	Groups				SEM	*p *value	Groups				SEM	*p *value
	CON	T1	T2	T3			CON	T1	T2	T3		
C12:0	0.1^AB^	0.25^B^	0.05^A^	Trace	0.03	0.008	0.03	Trace	Trace	Trace	–	–
C14:0	0.38^a^	0.47^b^	0.42^ab^	0.43^ab^	0.011	0.015	0.24^A^	0.28^A^	0.52^B^	0.6^B^	0.037	< 0.001
C14:1	0.04^A^	0.03^A^	0.08^B^	0.1^B^	0.007	< 0.001	trace	trace	trace	0.1	–	–
C15:0	0.1^A^	0.09^A^	0.07^B^	0.07^B^	0.003	0.001	0.08^A^	0.09^A^	trace	0.03^B^	0.009	0.006
C16:0	16.36^A^	16.6^A^	20.86^B^	21.49^B^	0.516	< 0.001	17.5^A^	17.44^A^	27.37^B^	26.1^B^	1.124	< 0.001
C16:1	1.88^A^	1.87^A^	3.08^B^	3.52^B^	0.162	< 0.001	0.52^A^	0.67^A^	2.76^AB^	4.54^B^	0.464	0.001
C17:0	0.19^A^	0.2^A^	0.12^B^	0.12^B^	0.009	< 0.001	0.38^A^	0.53^A^	0.13^B^	0.16^B^	0.038	< 0.001
C17:1	0.07^A^	0.03^B^	0.06^A^	0.06^A^	0.004	0.001	Trace	Trace	Trace	Trace	–	–
C18:0	7.23^A^	8.48^B^	7.00^A^	6.70^A^	0.168	< 0.001	23.42^C^	20.76^BC^	16.55^AB^	14.13^A^	0.916	< 0.001
C18:1 (trans-9)	0.04	0.02	0.03	trace	0.007	ND	0.1	0.03	0.07	0.09	0.011	ND
C18:1 (cis-9)	41.41^A^	39.47^B^	31.25^C^	31.08^C^	1.010	< 0.001	19.13^A^	24.64^AB^	31.38^BC^	33.228^C^	1.481	< 0.001
C18:2 n-6 LA	26.37^B^	25.7^B^	30.63^A^	30.5^A^	0.562	< 0.001	0.08^a^	0.1^b^	trace	trace	0.005	0.016
C20:0	0.12^B^	0.16^A^	0.08^C^	0.08^C^	0.007	< 0.001	20.04^B^	20.62^B^	13.93^A^	13.41^A^	0.929	0.001
C18:3 n-6	0.2	0.19	0.23	0.24	0.011	ND	0.162^AB^	0.2^B^	0.13^A^	0.15^A^	0.008	0.002
C20:1 n-9	3.3^A^	2.65^B^	3.2^A^	3.2^A^	0.070	< 0.001	0.11^ab^	0.07^a^	0.08^ab^	0.13^b^	0.009	0.049
C18:3 n-3 ALA	0.51^b^	0.57^a^	0.29^c^	0.29^c^	0.029	< 0.001	0.97	1.21	0.7	0.99	0.081	ND
C21:0	0.04	0.01	trace	trace	0.005	ND	0.47	0.58	0.31	0.69	0.063	ND
C20:2	0.27^B^	0.26^B^	0.33^A^	0.3^AB^	0.009	0.006	Trace	Trace	Trace	Trace	–	–
C22:0	Trace	Trace	Trace	Trace	-	-	1.05^B^	0.92^B^	0.36^A^	0.34^A^	0.074	< 0.001
C20:3 (all-cis-8,11,14)	0.26	0.25	0.28	0.28	0.008	ND	0.06	0.04	0.05	0.05	0.009	ND
C22:1	0.03	trace	0.03	0.02	0.006	ND	1.1^C^	0.71^B^	0.44^A^	0.35^AB^	0.072	< 0.001
C20:3 (all-cis-11,14,17)	1.23^AB^	1.55^A^	1.03^BC^	0.79^C^	0.072	< 0.001	0.22^C^	0.12^B^	0.02^A^	0.04^A^	0.018	< 0.001
C20:4 n-6	0.16^A^	0.2^A^	0.1^B^	0.09^B^	0.011	< 0.001	9.44^B^	7.795^B^	3.47^A^	2.83^A^	0.703	< 0.001
C20:5 n-3 EPA	0.08	0.09	0.06	0.05	0.006	ND	0.54^B^	0.41^B^	0.19^A^	0.15^A^	0.037	< 0.001
C24:1	0.29	0.28	0.34	0.26	0.014	ND	0.59^b^	0.36^ab^	0.17^a^	0.37^ab^	0.049	0.017
C22:6 n-3 DHA	0.26^a^	0.26^a^	0.16^b^	0.10^b^	0.018	< 0.001	3.14^C^	1.65^B^	0.67^A^	0.7^A^	0.230	< 0.001
Total SFA	24.01^C^	26.29^B^	28.61^A^	28.89^A^	0.440	< 0.001	18.72^A^	18.66^A^	30.92^B^	31.88^B^	1.478	< 0.001
Total MUFA	46.96^A^	44.34^B^	38.05^C^	38.24^C^	0.832	< 0.001	45.02^a^	48.07^a^	49.19^b^	48.90^ab^	0.578	0.029
Total PUFA	28.09^B^	27.65^B^	32.09^A^	31.84^A^	0.535	< 0.001	37.23^A^	33.80^A^	20.05^B^	19.33^B^	1.925	< 0.001
n-3 PUFA	2.06^B^	2.47^B^	1.54^A^	1.23^A^	0.113	< 0.001	4.97^C^	3.29^B^	1.59^A^	2.12^AB^	0.330	< 0.001
n–6 PUFA	27.25^B^	26.59^B^	31.58^A^	31.4^A^	0.567	< 0.001	22.83^B^	22.72^B^	15.0^A^	14.38^A^	1.063	< 0.001
n-6/n-3 PUFA	13.78^A^	10.81^A^	21.56^B^	26.72^B^	1.592	< 0.001	5.12^a^	7.51^ab^	10.49^b^	8.13^ab^	0.671	0.031

The "trace" value means ≤ 0.05 g/100 g; ND—not detected; CON—control group; T1—diet with upper dose of inorganic Se (0.5 mg/kg feed); T2—diet with upper dose of Se in the organic form (0.5 mg/kg feed); T3—diet with upper dose of Se in the form of nanoparticles (0.5 mg/kg feed); ^a,b,c^values denoted with various letters differ significantly at *p* ≤ 0.05; ^A,B,C^values denoted with various letters differ significantly at *p* ≤ 0.01.

The highest Se concentration in the breast muscle was determined in group T2 (*p* ≤ 0.01) fed a diet with SY (Table 2), whereas the lowest one in BM of the broilers from group T3. In the case of the liver samples, the highest Se concentration was determined in the CON group fed a diet supplemented with the recommended SS dose (0.3 mg Se/kg feed). The analysis of lipid oxidation indices showed significant (*p* ≤ 0.01) differences in their values depending on Se form. The TBA was significantly higher in the groups fed diets with SS (CON and T1), whereas the total antioxidative capacity measured with the radical scavenging method (DPPH, 1,1-diphenyl-2-picrylhydrazyl) and GSH was significantly higher in BM and liver of birds from groups T2 and T3.

**Table 2:** The effect of using increased doses of various forms of selenium on selenium content in tissues and oxidative indices.

Indices	Breast muscle						Liver					
	Groups				SEM	*p *value	Groups				SEM	*p *value
	CON	T1	T2	T3			CON	T1	T2	T3		
Selenium (mg/kg)	0.27^C^	0.37^B^	0.42^A^	0.12^D^	0.025	< 0.001	3.79^C^	2.9^B^	0.86^A^	0.49^A^	0.298	< 0.001
TBA (mg malonic aldehyde)	0.55^B^	0.54^B^	0.47^A^	0.30^AB^	0.091	< 0.001	2.08^a^	2.71^b^	2.36^ab^	2.04^a^	0.087	0.011
DPPH (%)	52.55^BC^	51.49^C^	55.83^AB^	56.12^A^	0.588	0.002	85.53^A^	85.71^AB^	86.42^BC^	86.56^C^	0.132	0.004
GSH (mmol-SH)	0.05^B^	0.04^B^	0.20^A^	0.25^A^	0.021	< 0.001	0.85^AB^	0.71^A^	0.89^B^	0.95^B^	0.026	0.004

*CON* control group, *T1* diet with upper dose of inorganic Se (0.5 mg/kg feed) , *T2* diet with upper dose of Se in the organic form (0.5 mg/kg feed) , *T3* diet with upper dose of Se in the form of nanoparticles (0.5 mg/kg feed); ^a,b^values denoted with various letters differ significantly at *p* ≤ 0.05; ^A,B,C^values denoted with various letters differ significantly at *p* ≤ 0.01.

The form of Se used in the diet for broilers had no significant (*p* ≥ 0.05) effect on PUFA/SFA ratio in BM, whereas its values in liver samples differed significantly (*p* ≤ 0.01) among the groups, with the highest PUFA:SFA ratio found in group CON (Table 3). The n-6/n-3 ratio was significantly higher in groups CON and T1, regardless of tissue examined. Fat from BM and liver of birds from groups and T featured a significantly (*p* ≤ 0.01) lower AI value, whereas BM fat from groups CON, T2, and T3, and liver fat from group T1 had a significantly (*p* ≤ 0.01) lower TI value. Sodium selenate administered to birds in the higher dose tested (0.5 mg/kg feed, group T1) had a negative effect on the TI value in BM, whereas in liver samples the same effect was observed upon diet supplementation with nano-Se. The values of hypocholesterolemic and hypercholesterolemic indices (h/H) were significantly (*p* ≤ 0.01) higher in the groups fed the SS-supplemented diet, both in breast muscle and liver. In addition, the dietary form of Se had a significant (*p* ≤ 0.01) effect on the values of DI (18), DI (16), total DI, thioesterase index (only in BM) and activity of Δ5-desaturase + Δ6-desaturase. The elongase activity (EI) differed significantly (*p* ≤ 0.05) between the groups; with the highest activity determined in both BM and liver of the birds from the groups fed the SS-supplemented diet. Contents of neutral and hypocholesterolemic acids (DFA) differed significantly between the tissues examined. The highest DFA concentration was determined in both BM and liver of the control birds. The value of the saturation index (S/P) and the content of hypercholesterolemic FA (HFA) differed significantly (*p* ≤ 0.01). The lowest values of these parameters were determined in the groups of birds fed the SS-supplemented diet.

**Table 3:** Lipid indices in the aspect of the human diet and enzymatic indices of fatty acid metabolism evaluated based on the fatty acid composition of breast muscle and liver.

Indices	Breast muscle						Liver					
	Groups				SEM	*p* value	Groups				SEM	*p* value
	CON	T1	T2	T3			CON	T1	T2	T3		
Desaturase 18 index (DI, 18)	85.30^B^	82.00^A^	81.69^A^	82.273^A^	0.322	< 0.001	52.07	76.96	Trace	Trace	9.212	ND
Desaturase 16 index (DI, 16)	10.22^A^	10.19^A^	12.85^B^	14.05^B^	0.390	< 0.001	43.93^A^	45.61^A^	5.93^B^	4.26^B^	4.721	< 0.001
Total DI	65.12^C^	62.26^B^	55.2^A^	55.1^A^	0.930	< 0.001	44.48^A^	48.64^A^	6.15^B^	4.18^B^	4.876	< 0.001
Elongase index (EI)	0.45^B^	0.51^C^	0.34^A^	0.31^A^	0.018	< 0.001	0.20^a^	0.05^ab^	0.04^a^	0.20^a^	0.023	0.013
Thioesterase index (TI)	43.26^AB^	35.76^A^	50.37^B^	50.22^B^	1.536	< 0.001	Trace	Trace	Trace	35.78	–	–
Δ5 + Δ6-desaturase index	6.03^BC^	7.62^C^	4.46^AB^	3.43^A^	0.394	< 0.001	22.58^B^	15.89^A^	10.20^A^	11.40^A^	1.280	< 0.001
PUFA/SFA ratio	1.15	1.05	1.12	1.11	0.017	ND	1.95^A^	1.78^A^	0.70^B^	0.61^B^	0.138	< 0.001
MUFA/SFA ratio	1.95^C^	1.69^B^	1.33^A^	1.32^A^	0.056	< 0.001	1.16	1.46	1.15	1.23	0.047	ND
UFA/SFA ratio	3.13^C^	2.74^B^	2.45^A^	2.43^A^	0.062	< 0.001	3.11^A^	3.25^A^	1.85^B^	1.83^B^	0.154	< 0.001
Saturation index (S/P)	0.31^C^	0.36^B^	0.4^A^	0.41^A^	0.009	< 0.001	0.01^A^	0.01^A^	0.04^AB^	0.07^B^	0.007	0.001
Atherogenicity index (IA)	0.23^C^	0.26^B^	0.32^A^	0.33^B^	0.009	< 0.001	0.01^A^	0.01^A^	0.05^AB^	0.08^B^	0.008	0.001
Thrombogenicity index (IT)	2.66^A^	3.43^B^	2.71^A^	2.35^A^	0.112	0.001	0.15^ab^	0.13^a^	0.27^ab^	0.72^b^	0.085	0.034
Desirable fatty acid (DFA)	81.86^C^	80.33^B^	77.15^A^	76.78^A^	0.462	< 0.001	82.35^A^	81.90^A^	69.33^B^	68.32^B^	1.539	< 0.001
HFA/hypercholesterolemic FA	16.33^A^	17.32^A^	21.32^B^	21.92^B^	0.529	< 0.001	0.69^A^	0.94^A^	3.33^B^	4.14^B^	0.381	< 0.001
h/H	4.60^C^	4.22^B^	3.29^A^	3.19^A^	0.129	< 0.001	127.35^A^	101.20^A^	22.69^B^	14.49^B^	12.123	< 0.001

The “trace” value means ≤ 0.05 g/100 g; *ND* not detected, *CON* control group, *T1* diet with upper dose of inorganic Se (0.5 mg/kg feed), *T2* diet with upper dose of Se in the organic form (0.5 mg/kg feed), *T3* diet with upper dose of Se in the form of nanoparticles (0.5 mg/kg feed); ^a,b^values denoted with various letters differ significantly at *p* ≤ 0.05; ^A,B,C^values denoted with various letters differ significantly at *p* ≤ 0.01.

## Discussion

Selenium is an essential element playing an important role in many physiological processes in animal bodies. It affects the appropriate growth, fertility, and immune responses^21^. In birds, Se is indispensable for the synthesis of selenocysteine, i.e. an amino acid being a constituent of selenoproteins involved in metabolic processes, like glutathione peroxidase (GSH-Px), thioredoxin reductase, and iodothyronine deiodinase^22^. Dietary components used in broiler feeding, including mainly cereals, can be deficient in Se mainly due to its shortages in the soil. Therefore, Se is supplemented in the poultry production to prevent its deficiencies in broiler chicken diet^23,24^. Broiler body demand for Se has been established at 0.15 mg/kg feed^25^. In turn, other authors recommend increasing this dose to 0.3 and 0.75 mg/ kg feed^26,27^. Given the ever increase rate of broiler chicken growth and, consequently, their faster body metabolism, it seems advisable to examine the impact of an increased diet supplementation with Se on their body protection against reactive oxygen species. This would help providing consumers with high-quality meat rich in fine-quality fat featuring a beneficial fatty acid profile^27,28^.

Supplementation of a diet for broilers chicken with Se affects its content in meat^29^, whereas Se form influences also its absorption, retention, and utilization in the bird bodies^30^. Selenium selenate is absorbed by the body via simple diffusion, whereas SY—via the active transport. This allows the birds to accumulate Se in tissues and then to use it for oxidative defense in the instance of intensified stress^31^. In the study by^29^, the highest Se concentration in BM and liver tissues was determined in the birds fed the SY-supplemented diet. These authors also showed that organic Se was more readily absorbed by the body compared to inorganic Se. In turn, in the present study, the highest Se concentration in BM was achieved in the birds from group T2 fed a diet with an increased dose of SY, whereas the highest Se concentration in the liver was determined in the birds from group T1 (SS, 0.5 mg/kg). This result also contradicts findings reported by^32^. The conducted study confirms that nano-Se shows higher bioavailability and lower toxicity risk than other Se forms^33^, especially SS. In view of the results of the present study, the recommendations for broiler diet supplementation with nano-Se at 0.3–0.5 mg/kg seem justified^26^. Furthermore, nano-Se added to a broiler diet in a upper recommended amount (0.5 mg/kg) may aid the proper functioning of the gastrointestinal tract. By this means, it can affect the proper development of birds’ immunity^34^, ensuring their high welfare and top quality of the finished product.

Given the increased risk of toxicity of inorganic forms due to their easy accumulation in tissues, there is a need for research on other forms of Se, which will provide important information for modern poultry production for the sake of both chickens welfare food safety for humans. 100 g of breast muscle from chickens fed a diet with elevated levels of nano-Se in feed (0.5 mg/kg) can provide 17% of the Recommended Daily Intake (RDI of Se = 70 µg/day) for an adult human^35^. Consumption of breast muscle from T2 chickens provides up to 60% of the RDI. In contrast, Se tends to concentrate strongly in the liver. Livers from chickens fed CON, T1, (inorganic Se) diets had a successively four, five times higher recommended RDI, while those from the T2 group had more than one time higher RDI, which may pose a risk to human health. Livers from T2 chickens could potentially provide up to 70% of the RDI of this element (per 100 g of chicken liver). Appropriate supplementation with nano-Se at an increased dose (0.5 mg/kg) can result in a final product with increased nutritional value, referred to as functional food. This confirms that meat and livers enriched with nano-Se in this manner are a valuable and safe source of Se that can contribute to improved consumer health, while maintaining the need for this element in broiler chickens and ensuring their normal growth, health and thus welfare levels. The use of nano-Se in the diet of chickens at the maximum dose of 0.5 mg/kg is allowed by the European Union regulations^36^, as it brings mutual benefits for both the chickens and the quality of the raw material obtained from the birds. Also, nano-Se seems effective in modulating the fatty acid profile of broiler chicken meat and giblets. These findings indicate that the supplementation of broiler diet with the nanosized form of Se at the dose as in the present study is safe as its other forms in terms of enriching meat with Se, which may be important for the consumer health. In addition, nano-Se protects the liver of broiler chickens against the detrimental effect of Cr(VI)^37^. In their study^38^ supplemented a broiler diet with SY in a dose of 0.5 mg/kg and found that it allowed producing meat enriched with selenium at 0.256 mg/kg and enriched with n-3 FA up to 6.71% of total FA. The comparison of results obtained for the SY-supplemented group demonstrated that it was characterized by the highest Se concentration in BM (0.42 mg/kg) and by a substantially lower content of the n-3 fatty acids (1.54% of total FA). The analysis of the present study results in terms of the human diet recommendation shows that supplementing broiler diet with a higher SS dose (0.5 mg/kg feed) allows achieving the highest Se concentration in BM, the best n-6/n-3 ratio, and the highest ALA concentration.

Broiler diet supplementation with SS resulted in lower contents of C14:0 and C:16 in BM and in the liver, compared to the other additives tested. The above findings are essential from the standpoint of human nutrition because these FA can be potential promoters of cardiovascular diseases^39^. Broiler diet supplementation with SS (groups CON and T1) caused also a clear tendency for the increased concentration of n-3 PUFAs, compared to the other Se forms tested. This correlation can be explained by the greater protection ensured by inorganic Se and, thus, by reduced degradation of PUFAs in oxidative processes^40^. The latter can be ascribed to the activity of glutathione peroxidase (GPx), i.e. a selenium-dependent enzyme the main function of which is to eliminate free radicals and, consequently, to protect FA, including especially PUFAs. Selenium is an important component of GSH-Px, whereas Se supplementation can enhance mRNA GSH-Px1 expression in the liver of chickens^41^. In the present study, Se used in the form of SY and nano-Se was found to significantly affect PUFA accumulation in BM, and to diminish it in the liver. This is linked to the functions of BM and liver. Breast muscle accumulates PUFA mainly in lipids whereas liver is responsible for their distribution in the body. The increased level of PUFA in a diet, especially of n-3 PUFA, can lead to lipid peroxidation and, consequently, to the impairment of liver functions^42,43^. We evidenced that the use of other than SS forms of Se had a more beneficial effect on the proper lipid metabolism in birds.

The n-3 and n-6 FA belong to two different families and cannot be synthesized by the organisms of mammals and birds. The excess of fatty acids from one family impairs the metabolism of those from the other group; hence, it is essential to maintain the optimal n-6/n-3 ratio. The use of SS in the chickens diet in the present study was found to significantly modify the FA composition, by ensuring more beneficial n-6/n-3 ratio, which is recommended in the prevention of the ischemic heart disease^44^. The disturbed n-6/n-3 ratio leads to the overproduction of proinflammatory eicosanoids, which stimulate the synthesis of cytokines and acute phase proteins, which in turn are triggers of such diseases as neoplasms, cardiovascular diseases, atherosclerosis, obesity, type 2 diabetes, or Alzheimer’s disease^45,46^. In turn, lauric (C12:0), myristic (C14:0), and palmitic (C16:0) acids strongly correlate with the risk of the incidence of atherosclerosis, obesity, or ischemic heart disease^47,48^, whereas the degree of saturation with these acids is considered to be a parameter of food quality assessment^49^. In the present study, dietary supplementation with SS significantly (*p* ≤ 0.01) diminished the content of saturated fatty acids and increased ALA content in BM, which is deemed important from the human nutrition perspective.

The TBARS value (mg MDA/kg sample) indicates the status of lipid oxidation in different tissues, whereas DPPH serves to measure the capability for scavenging free radicals^50,51^. The values of atherogenicity (AI) and thrombogenicity (IT) indices ought to be as low as possible. The lower their values are, the smaller is the probability of atherosclerosis incidence and blood clots development in humans^52^.

Unlike the AI and TI indices, the values of the hypocholesterolemic and hypercholesterolemic indices (h/H) need to be as high as possible to protect a consumer against hypercholesterolemia, which is a risk factor of the atherosclerotic syndrome^53^. In the present study, liver samples, having high contents of lipids and minerals that elicit prooxidative effects, had higher TBARS values compared to BM The addition of selenates (0.3 mg/kg) and nano-Se (0.5 mg/kg) to diet caused a significant (*p* ≤ 0.05) decrease in the content of lipid oxidation products in liver tissues, i.e. to 2.08 and 2.04 μg MDA/g, respectively. Also^54^ observed the protective effect of Se on liver tissues against changes triggered by free radicals. They showed that diet supplementation with nano-Se significantly reduced the content of lipid oxidation products, from 0.55 in group C to 0.30 mg MDA/g in the amended group.

Delta-9 desaturase catalyzes the transformation of medium-chain and long-chain SFA into individual MUFA, i.e. C16:0 and C18:0 as well as C16:1 and C18:1, respectively^55^. Delta-9 desaturase activity depends on the diet and age of birds^56^, but also on the form of Se implemented in the diet, as indicated in the present study. The increase in delta-9 desaturase activity in the groups fed the SS-supplemented diet caused a decrease in contents of C16:0 and C18:0 FA in favor of C16:1 and C18:1 fatty acids in BM The organisms of both mammals and birds are incapable of synthesizing essential PUFA, like LA and ALA from acetyl-CoA, but can transform them into more unsaturated long-chain essential FA when they are provided with diet. Transformations of these acids are catalyzed by, among other things, desaturases. The Δ5 + Δ6-desaturase index is used to assess birds’ capability to synthesize long-chain FA from LA and ALA^57^. The higher value of the Δ5 + Δ6-desaturase index indicates a higher effectiveness of long-chain FA synthesis. Broiler chickens are capable of synthesizing DHA and EPA from ALA, and this synthesis is catalyzed by desaturase and elongase^58^. In the present study, an increased DHA content was determined in both BM and liver of the birds fed the SS-supplemented diet. The EPA content did not differ significantly (*p* ≥ 0.05) between BM samples, but significant (*p* ≤ 0.01) differences were noted in its content between the liver samples. The increased contents of EPA and DHA are confirmed by a significantly higher activity of Δ5 + Δ6-desaturase in groups CON and T, which allows concluding that Se addition in the form of SS had a significant effect on the effectiveness of long-chain FA synthesis.

## Conclusion

The study showed that selenium supplementation at a dose of 0.5 mg/kg feed significantly influences selenium concentration in breast muscle and liver, modifications of fatty acid profile, oxidative indices and lipid metabolism. The application of nano-Se in the amount of 0.5 mg/kg of feed is characterized by a significantly higher content of PUFAs and protection of lipids against the action of reactive oxygen species, with its high bioavailability and low toxicity for the chicken organism. The use of increased doses of selenium in feed is a response to the increasingly rapid growth rate and orgasmic metabolism of chickens. This will help to provide consumers with a high quality product rich in good quality fats.

## Methods

### Animals and diets

The experiment was carried out with 300 Ross 308 chicken broilers randomly allocated to 4 experimental groups, in 5 replications, 15 birds per replication. Broilers were reared under standard conditions for 42 days. They had free access to water, and were kept under a controlled light cycle. For the first 10 days, all birds were fed the same starter diet balanced to meet their nutritional demands (Table 4). On day 11 of life, they started to receive respective diets (Table 5). Experimental groups differed in terms of selenium form implemented in the diet, i.e.:

**Table 4:** Starter diet.

Analytical components	Starter diet
Crude protein, %	22.00
Crude fiber, %	3.00
Crude fat, %	4.7
Crude ash, %	5.6
Lysine, %	1.26
Methionine, %	0.58

**Table 5:** Experimental diet.

Analytical components	CON	T1	T2	T3
Crude protein, %	20.46	20.74	20.78	20.89
Crude fiber, %	3.05	3.32	3.20	5.89
Crude fat, %	6.90	6.68	6.60	6.44
Crude ash, %	3.10	2.42	2.07	6.41
Lysine, %	1.38	1.42	1.41	1.39
Methionine, %	0.43	0.43	0.46	0.43

*CON* control group, *T1* diet with upper dose of inorganic Se (0.5 mg/kg feed) , *T2* diet with upper dose of Se in the organic form (0.5 mg/kg feed) , *T3* diet with upper dose of Se in the form of nanoparticles (0.5 mg/kg feed).

CON—control group—diet meeting nutritional demands of Ross 308 broilers with the basic (recommended) dose of inorganic Se (SS—0.3 mg/kg feed),

T1 (SS)—diet with upper dose of inorganic Se (SS 0.5 mg/kg feed),

T2 (SY)—diet with upper dose of Se in the organic form (SY, commercial preparation) (0.5 mg/kg feed),

T3 (Nano-Se)—diet with upper dose of Se in the form of nanoparticles (commercial preparation) (0.5 mg/kg feed).

### Sampling procedures

Forty chickens were chosen (10 birds from each treatment; 2 birds for each replicate) for slaughter at the age of 42 days of life that had a body weight similar to the group mean. Tissues samples were collected for biochemical and antioxidant analysis**.**

### Selenium content

Determination of Se content in breast muscle and liver was performed according to the PB-28/LF method in an accredited laboratory (PCA Accreditation Certificate No. AB 1095 Issue No. 19 dated January 1, 2022). Preparation of samples according to PN-EN 13804 standard, followed by mineralisation (MARS 5) was implemented according to PN-EN 13805 standard. Determining Se content in tissues was performed using the ICP-MS technique (Thermo XSERIES 2 system). The results were compared to the ICP multielement standard solution (19 elements in dilute nitric acid, CERTIPUR, 115474 Merck Millipore).

### Antioxidant capacity

2-Thiobarbituric acid (TBA) in tissues was determined by the extraction method according to^59^, which involved measuring the absorbance of color solution, the color of which developed as a result of the reaction between fat oxidation products (mainly malonaldehyde) and TBA. Approximately 2 g of fat was weighed into a centrifuge tube to the nearest 0.01 g, to which 5 cm^3^ of 10% trichloroacetic acid was added; then the mixture was triturated for 2 min with a glass rod. Next, 5 cm^3^ of 0.02 molar TBA solution was added and the sample was triturated again for 2 min and centrifuged for 10 min at 4000 rpm. After centrifugation, the solution was filtered into a glass tube, and after sealing the opening with polythene sheeting, the color was developed for 24 h at room temperature. Afterwards, samples were collected for colorimetric determination. The absorbance was measured using a Hitachi U-1100 spectrophotometer at 532 nm against the reagent blank. The reagent blank was prepared by adding 5 cm^3^ of 10% trichloroacetic acid and 5 cm^3^ of 0.02 molar TBA solution to a glass tube.

Measurements for radical scavenging activity in analyzed tissues were performed by routine assay procedure^60^ using a synthetic DPPH radical (1,1-diphenyl-2-picrylhydrazyl). Folin–Ciocâlteu reagent was used as an oxidizing reagent, and all the chemicals were purchased from SIGMA-ALDRICH CHEMIE GMBH (Munich, Germany) in the highest available purity.

Glutathione (GSH) concentration was determined in tissues by means of the OXISRESEARCH BIOXYTECH GSH/GSSG—412™ test (Foster City, CA, USA). Before the analysis, the samples were frozen with the addition of M2VP (1-methyl-2-vinyl-pyridium trifluoromethanesulfonate) at a temperature of 80 °C. The released, reduced GSH was determined in accordance with the detailed instruction provided by the kit’s producer. The absorbance reading (λ412) and the measurement of reaction kinetics were performed using the microplate reader Synergy 4 (BIOTEK; Winooski, VT, USA). The results were calculated using Gen5 software (BIOTEK). GSH concentration was expressed in thiol groups (mmol-SH groups).

### Fatty acid composition

Total lipids from tissues were extracted following the procedure described by Folch et al.^61^. The fatty acids profile was determined with the gas chromatograph with FID detector according to PN-EN ISO: 5509, PN-EN ISO: 5508 as previously by Ciemniewska-Żytkiewicz^62^. Used Restek-2330 capillary column, 105 m, 0.25 mmID, 0.2 µm df (90% biscyanopropyl/10% phenylcyanopropyl polysiloxane). Initial column temperature 100 °C for 4 min. Then incremented to 240 °C at 3 °C/min. The final temperature was kept to a minimum until the elution of the last chromatographic peak. FID detector temperature 300 °C. H_2_ flow 30 ml/min in FID detector, airflow 350 ml/min in FID detector, N_2_ flow (make-up) 15 ml/min in FID detector. A single-point detector calibration was used for all fatty acids determined based on the standard. During calibration, the RF (response factor) was determined for each fatty acid methyl ester. Calibration curve using certified BCR-162R reference material. The basic standard contains 37 fatty acids with the same or similar composition to the standard ‘Supelco 37 Component Fame Mix’, undissolved or dissolved in hexane (stored according to the manufacturer's instructions). Determining the fatty acid profile was performed in an accredited laboratory (PCA Accreditation Certificate No. AB 439 Issue No. 18 dated August 2, 2019).

### Fatty acid metabolism indices

Desaturation indices were computed by referring the percentage content of the product to the percentage content of the precursor, as follows^63^:

$$\mathrm{D}I \left(18\right) {:}\, {\Delta }^{9}-desaturase \, \left(18\right) \, index=100\times \frac{C18{:}1}{C18{:}1+C18{:}0}$$

$$DI \left(16\right) {:} \,{\Delta }^{9}-desaturase \, \left(16\right) \, index=100\times \frac{C16{:}1}{C16{:}1+C16{:}0}$$

$$Total\,\, DI\,\, {\Delta }^{9}-desaturase \, index=100\times \frac{C16{:}1+C18{:}1}{C16{:}1+C16{:}0+C18{:}1+C{:}18{:}0}$$

The elongase index (EI) and thioesterase index (TI) were calculated as follows^64^:

$$Elongase\,\, index \, \left(EI\right)= \frac{C18{{:}}0}{C16{:}0}$$

$$Thioesterase \,\,index \, \left(TI\right)= \frac{C16{:}0}{C14{:}0}$$

The activity of Δ5-desaturase + Δ6-desaturase was determined using the following equation^65^:

$${\Delta }^{5}-\mathrm{desaturase}\,+ {\Delta }^{6}-\mathrm{desaturase}=100\times \frac{\mathrm{C}20{:}2\,\mathrm{ n}6+\mathrm{C}20{:}4\,\mathrm{ n}6+\mathrm{EPA}+\mathrm{C}22{:}5\,\mathrm{ n}3+\mathrm{DHA}}{\mathrm{C}18{:}2\,\mathrm{ n}6\left(\mathrm{LA}\right)+\mathrm{ALA}+\mathrm{C}20{:}2\,\mathrm{ n}6+\mathrm{C}20{:}4\mathrm{ n}6+\mathrm{EPA}+\mathrm{C}22{:}5\,\mathrm{ n}3+\mathrm{DHA}}$$

### Estimation of health indices

The established fatty acid profile enabled calculating the n-3/n-6 ratio, PUFA/SFA ratio, monounsaturated fatty acids (MUFA)/SFA ratio, and UFA/SFA ratio. The saturation index (S/P) and the atherogenicity (AI) and thrombogenicity (TI) indices were computed as follows^47^:

$$Saturation\,\, index \, (S/P)= \frac{C14{:}0+C16{:}0+C18{:}0}{MUFA+PUFA}$$

$$Atherogenicity\,\, index \, (AI)= \frac{C12{:}0+4\times C14{:}0+C16{:}0}{MUFA+PUFA}$$

$$Thrombogenicity\,\, index \, (TI)= \frac{C14{:}0+C16{:}0+C18{:}0}{\frac{MUFA+PUFA}{2+3\,\times n3 PUFA+\frac{n3}{n6}}}$$

The DFA, HFA, and h/H ratios were calculated as follows^66^:

$$DFA{:}\,desirable \,\,fatty\,\, acid=UFA+C18{:}0$$

$$HFA/hypercholesterolemic \, FA (C12{:}0+C14{:}0+C16{:}0)$$

$$\frac{h}{H} = \frac{{hypocholesterolemic{\mkern 1mu} \;(C18{:}1 + PUFA)}}{{{\mkern 1mu} hypercholesterolemic\;ratio\;(C14{:}0 + C16{:}0)}}$$

### Ethical approval

All procedures in the present study were performed in accordance with the principles of the European Union and Polish Law on Animal Protection. No action involving pain or suffering was practiced. All applicable institutional guidelines for the care and use of animals were followed. The experimental procedures carried out in this study were approved by the 2nd Local Ethical Committee for Animal Experiments at the Warsaw University of Life Sciences (1 September 2021). The study was carried out in compliance with the Animal Research: Reporting of in Vivo Experiments (ARRIVE) guidelines.

### Statistical evaluation

The Principal Component Analysis (PCA) was conducted for tentative data exploration using the STATISTICA 13.0 software. Mean values of fatty acid contents in the analyzed samples were processed using the PS IMAGO PRO 6.0 statistical package employing one-way analysis of variance (ANOVA). Tukey’s test was used to determine the significance of differences between the examined groups. In turn, Student's t-test was used to compare two groups.

The above-mentioned analyses were expected to provide an explicit answer to the question whether the use of various selenium forms in a diet positively affects the modification of fatty acid profile in selected tissues of chicken broilers. At the same time, they would enable establishing the most appropriate form of selenium to be used in broiler diet in order to improve meat quality and, thereby, to enrich the human diet with fine-quality fats.

## Data Availability

The datasets utilized and analyzed during this investigation are available upon reasonable request from the corresponding author.
